# Single-cell mechanistic studies of radiation-mediated bystander effects

**DOI:** 10.3389/fimmu.2022.849341

**Published:** 2022-10-25

**Authors:** Xueqiong Han, Yixuan Chen, Nan Zhang, Chengyu Huang, Guangyao He, Ting Li, Mengxin Wei, Qiong Song, Shaowen Mo, Yufeng Lv

**Affiliations:** ^1^ Department of Oncology, The Fifth Affiliated Hospital of Guangxi Medical University, Nanning, China; ^2^ Department of Basic Science, YuanDong International Academy Of Life Sciences, Hong Kong, China; ^3^ Department of Otolaryngology-Head and Neck Surgery, The First Hospital of Guangxi Medical University, Nanning, China; ^4^ Department of Oncology, Foresea Life Insurance Guangxi Hospital, Nanning, China

**Keywords:** radiotherapy, ferroptosis, bystander effect, reprogramming, cell communication

## Abstract

Ionizing radiation (IR) has been widely used in the diagnosis and treatment of clinical diseases, with radiation therapy (RT) being particularly rapid, but it can induce “bystander effects” that lead to biological responses in non-target cells after their neighboring cells have been irradiated. To help clarify how radiotherapy induces these effects, To help clarify how radiotherapy induces these effects, we analyzed single-cell RNA sequencing data from irradiated intestinal tissues on day 1 (T1 state), day 3 (T3 state), day 7 (T7 state), and day 14 (T14 state) after irradiation, as well as from healthy intestinal tissues (T0 state), to reveal the cellular level, molecular level, and involvement of different time irradiated mouse intestinal tissues in biological signaling pathways. In addition, changes in immune cell subpopulations and myeloid cell subpopulations after different radiation times were further explored, and gene regulatory networks (GRNs) of these cell subpopulations were constructed. Cellular communication between radiation-specific immune cells was explored by cell-to-cell communication events. The results suggest that radiotherapy trigger changes in immune cell subsets, which then reprogram the immune ecosystem and mediate systemic bystander effects. These radiation-specific immune cells participate in a wide range of cell-to-cell communication events. In particular, radiation-specific CD8+T cells appear to be at the core of communication and appear to persist in the body after recovery from radiotherapy, with enrichment analysis showing that radiation-specific CD8+ T cells are associated with ferroptosis. Thus, radiation-specific CD8+ T cells may be involved in cellular ferroptosis-mediated adverse effects caused by RT.

## Introduction

Ionizing radiation (IR) has been widely used for the diagnosis and treatment of clinical diseases, and radiation therapy (RT) has developed particularly rapidly. Over the past 20 years, radiation therapy has been one of the most effective treatments in cancer therapy and plays a central role in 25% of all cancer treatments (1–3). As the number of patients receiving radiation therapy has steadily increased, the incidence of radiation therapy-related complications has increased accordingly (4, 5). Between 6% and 78% of survivors after long-term radiation have been reported to suffer from complications that result in a reduced quality of life for patients (6). The most common side effect of this therapy is intestinal mucositis caused by damage to normal intestinal epithelial cells. Radiation therapy-induced intestinal mucosal atrophy and ulceration impede the renewal of basal epithelial cells (radiation enteritis) (7, 8).

IR is generated by special devices whose mechanism of action is to induce double-stranded DNA damage, which ultimately leads to cell death (9). At the molecular level, when tumor cells are directly exposed to radiation beams, the atoms of the tumor cells are ionized, which excites electrons, and these secondary electrons directly damage the nuclear DNA of the tumor cells, and the incomplete repair of these damaged DNA leads to several modes of tumor cell death, such as apoptosis, necrosis, autophagy, mitotic catastrophe, or replicative senescence (9–11). However, recent studies have found that while the direct action of IR on cellular DNA is thought to be the primary mechanism of tumor cell death, it also exerts systemic antitumor effects through other mechanisms, such as the stimulation of the body’s immune system and the “bystander effect,” implying that radiation therapy has not only local effects on target tumor tissue but also on distant tumor tissue. This is thought to be an important factor in the adverse effects of radiotherapy (12). Radiation-induced bystander effect (RIBE) is the ability of cells exposed to external effects to transmit the manifestation of the effect to cells not directly exposed to external effects; where RIBE mainly has cell death, gene mutation, chromosomal instability, and other similar biological changes in unirradiated normal cells and irradiated cells (13). Cells exposed to IR (target cells) can communicate their DNA damage response state to cells not directly irradiated (bystander cells) (14). Irradiated cells appear to fire signals to non-irradiated cells, such as through gap junctions, intercellular communication, and mediated transfer mechanisms (15). However, it is not clear how irradiation induces bystander effects.

In the present study, we investigated the potential role of immune cells in RT-induced bystander effects. We used single-cell RNA sequencing data from irradiated tissues to examine changes in immune cell subpopulations and analyzed how these changes might relate to cell-cell communication and gene regulatory networks. This allowed us to identify potential pathways through which IR can trigger bystander effects. Our results suggest that radiation reprograms the immune cell ecosystem.

## Results

### Radiotherapy reprograms the cellular level of intestinal tissues

**Figure 1:**
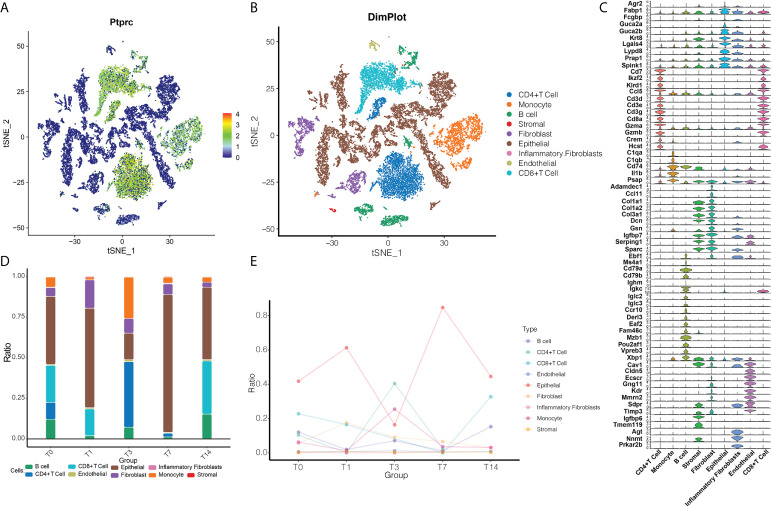
RT reprograms intestinal tissues at the cellular level. **(A)** The single-cell map based on CD45. Blue indicates that the cells were negative for CD45; green, that the cells were positive for CD45. **(B)** Cell ecosystem after radiation exposure. Different colors represent different cell types. **(C)** Expression of marker genes in different cells undergoing radiotherapy reprogramming. **(D)** Cell ecosystem after radiation exposure. The colors of the columns represent different cell types. **(E)** Changes in the ecosystem over time after radiation exposure.

In order to explore radiation-induced changes in cellular and molecular states at the single-cell level, we first used data from individual irradiated cells to construct an immune cell ecosystem. After defining cell clusters, we plotted cells expressing the common leukocyte antigen CD45 in a single-cell map (**Figure 1A**). After irradiation, the ecosystem consisted mainly of CD4+ T cells, CD8+ T cells, monocytes and epithelial cells (**Figure 1B**). Known cell marker genes were specifically expressed in all these cell type clusters (**Figure 1C**).

We then further compared how the cell abundance levels changed with time after RT (**Figures 1D** **, E**). The T1 state was found to have a lower number of immune cells compared to the T0 state, indicating an inflammatory state. By the T3 state, CD4+ T cells became more abundant, when the number of monocytes and endothelial cells was higher than in the T0 state, but the number of epithelial cells was lower than in the T0 state. By the T7 state, the abundance of B cells, CD4+ T cells, CD8+ T cells, and monocytes decreased. By the T14 state, the abundance of B cells, endothelial cells, stromal cells, epithelial cells and fibroblasts was similar to the T0 state, while the levels of monocytes and CD4+ T cells were lower and the levels of CD8+ T cells were higher than the T0 state, presumably T14 was the recovery state. Therefore, we can tentatively conclude that radiation reprogrammed the immune microenvironment of the intestinal tissues.

### RT-induced changes in lymphocyte subpopulations in intestinal tissues

**Figure 2:**
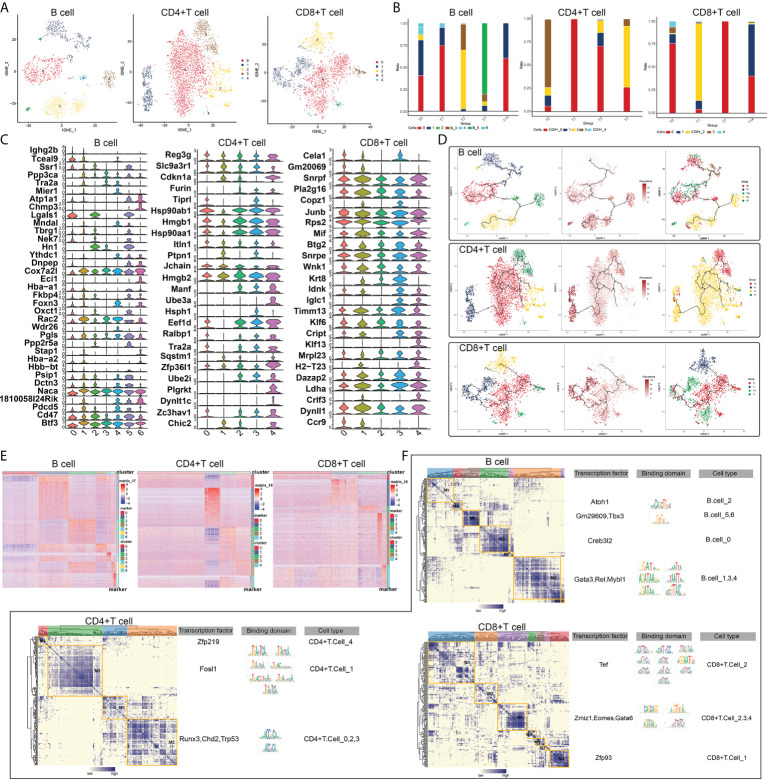
RT-induced changes in lymphocyte subpopulations in intestinal tissues. **(A)** Radiation reprograms the subset of lymphocyte immune cells. **(B)** Changes in abundance of lymphoid immune cell subsets. The colors of the different columns represent the changes in lymphocyte immune cells at different times after radiotherapy. **(C)** Radiation reprograms the expression of marker genes in a subset of lymphocyte immune cells. **(D)** Radiation reprogramming of immune cell subsets in the lymphatic system with increasing time after radiation exposure. **(E)** Expression of lymphoid immune cell genes in different subgroups. **(F)** Regulatory sub-modules of lymphoid immune cell subsets. B cell regulators were divided into seven modules; CD4+T cell regulators, four modules; and CD8+ T cell regulators, seven modules. The right side of the module diagram shows the regulator motif of lymphoid immune cells.

There were significant changes in the levels of lytic immune cells, such as B cells, CD4+ T cells and CD8+ T cells, *in vivo* after RT at different times, and we can consider these as radiation-specific immune cells. Subpopulations of B cells, CD4+ T cells and CD8+ T cells were identified, a total of six subpopulations of B cells were captured, five subpopulations of CD4+ T cells were captured, and six subpopulations of CD8+ T cells were also captured (**Figure 2A**), it is noteworthy that the subpopulations of B cells, CD4+ T cells and CD8+ T cells all changed significantly at different RT times (**Figure 2B**) in which again the heterogeneity of radiation-specific immune cell subpopulations is illustrated, suggesting that they may contribute to the adverse response of the intestinal tissues after RT. In addition, the expression of marker genes of radiation-specific immune cell subpopulations was further explored (**Figure 2C**). The evolutionary trajectory of the radiation-specific immune cell subpopulation and the expression of the pseudo-time-related genes were explored by mimetic time series analysis (**Figures 2D** **, E**).

Since gene expression is regulated by transcription factors, we constructed a GRN of radiation-specific immune cell subpopulations and hierarchically clustered the regulatory factors according to the Conjugation Specificity Index (CSI) to rank the importance of regulatory factors and mitigate the effects of non-specific interactions, and our results showed that CD4+ T cells were mainly regulated by ZFP219, FOSL1, RUNX3 and CHD2; B cells were mainly regulated by ATOH1, GM29609, TBX3 and CREB3L2; and CD8+ T cells were mainly regulated by TEF, ZMIZ1 and GATA6 (**Figure 2F**). These TFs were composed into different modules, which in turn regulated the RT-induced expression of specific genes in radiation-specific immune cells in intestinal tissues.

### Changes in bone marrow immune cells in RT-induced intestinal tissues

**Figure 3:**
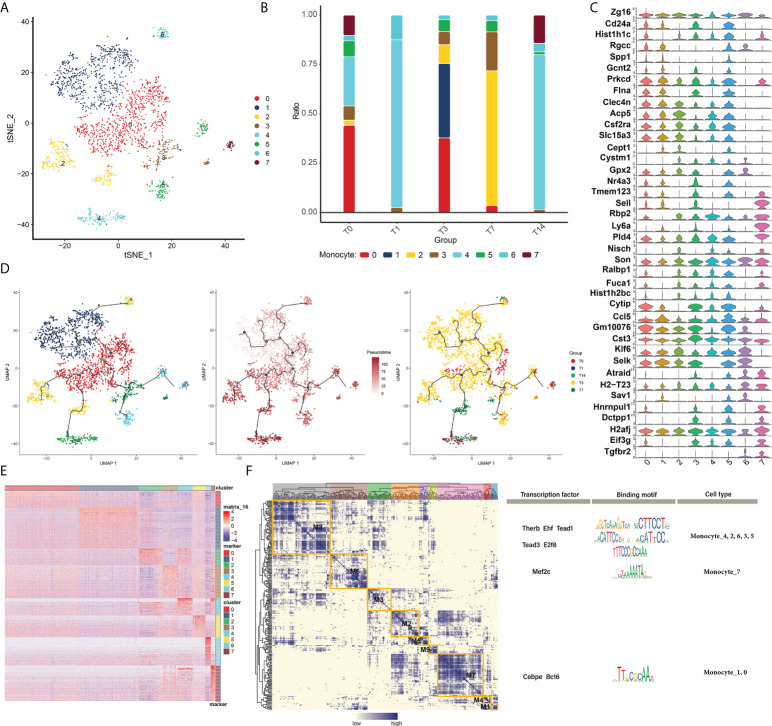
Changes in myeloid immune cells in RT-induced intestinal tissues. **(A)** Radiation reprograms the subset of myeloid immune cells. **(B)** Changes in the abundance of myeloid immune cells subsets. The colors of the different columns represent the changes in the immune cells at different times after radiotherapy. **(C)** Radiation reprograms the expression of marker genes in a subset of myeloid immune cells and monocytes. **(D)** Radiation reprograms the immune cell subsets of the myeloid lineage with increasing time after radiation exposure. **(E)** Expression of myeloid immune cell monocyte genes in different subgroups after radiation exposure. **(F)** Regulatory sub-module of myeloid immune cell monocyte subsets. Regulators of monocytes were divided into eight modules, and the motif of the regulator is shown on the right.

Some myeloid immune cells, such as monocyte subpopulations, were changed after RT. Subpopulation identification of monocytes yielded a total of eight subpopulations (**Figure 3A**). The changes in abundance of different subpopulations at different RT times were analyzed to observe the changes in cell levels after different times of RT (**Figure 3B**). And the expression of marker genes of monocyte subpopulations was plotted in the violin atlas (**Figure 3C**). In addition, the evolutionary trajectory of the monocyte cell subpopulation and the expression of the proposed time-related genes were explored by mimetic time series analysis (**Figures 3D** **, E**).

We constructed the GRN of monocytes (in order to better search for key regulators that drive the maintenance of cellular state behavior, as shown in the results, and it is known that the GRN branched by TFs such as THERB, EHF, TEAD1, TEAD3, E2F8 and MEF2C is organized into nine modules **Figure 3F**), which in turn regulate RT-induced expression of specific genes in specific monocyte cells in intestinal tissues.

### Global regulatory network of microenvironmental immune cells in RT-induced intestinal tissues

**Figure 4:**
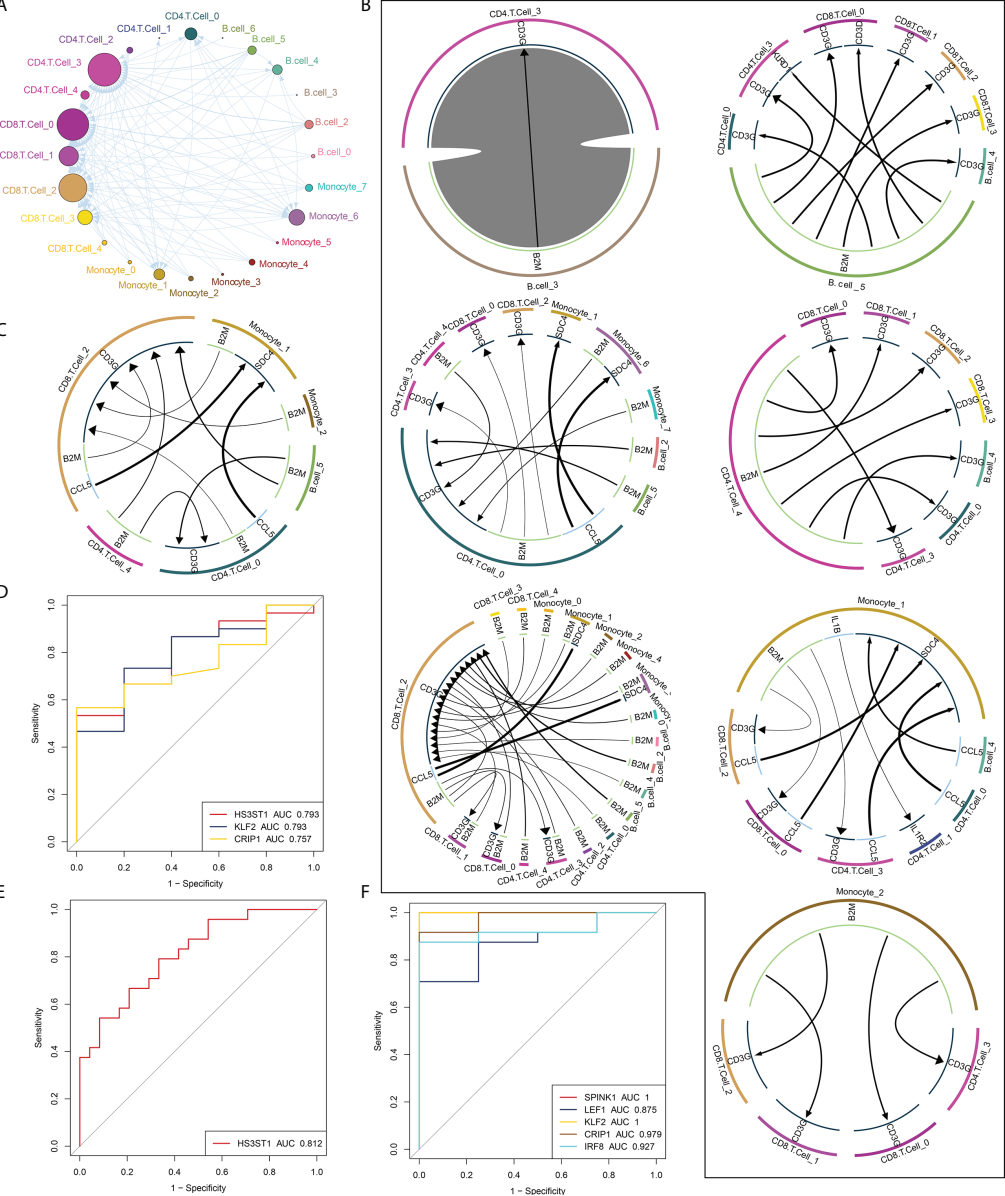
Global regulatory network of microenvironmental immune cells in RT-induced intestinal tissues. **(A)** Radiation reprogramming of the immune cell communication network. Different colors represent different cell types. **(B)** The communication network between immune cells and other cells. The outer circle of the Circos diagram shows the cell type, and the inner circle shows the details of each interacting ligand-receptor pair. The lines and arrows in the Circos diagram are scaled to indicate the relative signal intensity of the ligand and receptor, respectively. **(C)** Radiation-specific immune cell communication network. **(D–F)**. Composite ROC curves showing the diagnostic efficiency of core cell marker genes. Different curves represent different genes, and the closer the AUC value is to 1, the stronger the diagnostic ability of the gene. ROC, receiver operating characteristic; AUC, area under the ROC curve.

To determine the mechanisms of intercellular regulation of the microenvironment in intestinal tissues, we analyzed the intercellular communication of immune cells to explore differentially expressed ligands and receptors in different cell types, which allowed us to construct a comprehensive regulatory network that may help decipher the effects of RT on the immune microenvironment in intestinal tissues. We first established a communication network between all immune cell subpopulations of RT (**Figure 4A**), and in addition, the communication network between radiation-specific immune cells and other immune cells (**Figure 4B**) and the cellular communication network between radiation-specific immune cells (**Figure 4C**) were constructed, respectively. By constructing the above intercellular communication network, we can know that radiation-specific CD8+ T cells are at the core of the dysregulated radiation immune microenvironment.

Based on peripheral blood data (GSE21240, GSE55953, GSE65292) from irradiated patients, we found that some markers in these core cells can predict radiation-induced damage through ROC curve analysis (**Figures 4D** **–F**), including HS3HTI, KLF2 and CRIP1.

### RT-induced intercellular communication of microenvironmental immune cells in intestinal tissues

**Figure 5:**
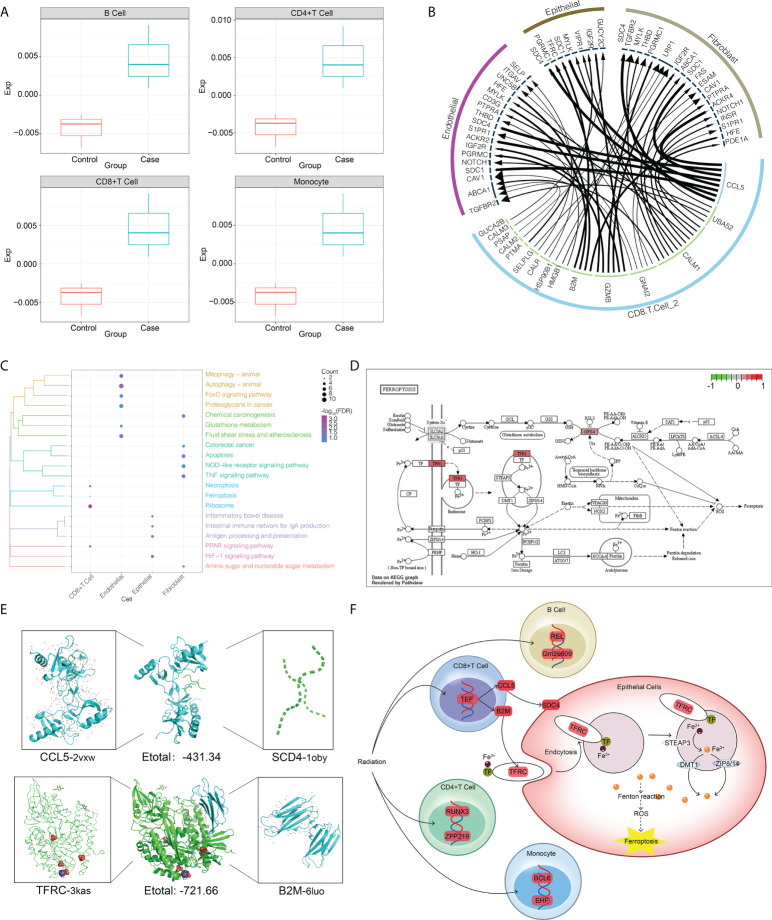
RT-induced intercellular communication of microenvironmental immune cells in intestinal tissues. **(A)** The abundance of radiation-specific immune cells was significantly higher than that of the control group. **(B)** The interaction of radiation-specific CD8+T cells with endothelial cells, epithelial cells, and fibroblasts. The outermost circle is the cell type, and the inner circle is the surface receptor of the cell. **(C)** Genes regulated by radiation-specific immune cells CD8+ T cells activate the iron death pathway in epithelial cells. Where red represents upregulation of gene expression. **(D)** Pathways significantly enriched in endothelial cells, epithelial cells and fibroblasts after radiation. The clustering tree of the pathway is shown on the left. The size of the bubble represents the number of genes involved in the pathway, and the color of the bubble represents the P value of the pathway. **(E)** Three-dimensional structures showing molecular docking of B2M with CCL5 or TFRC. **(F)** Mechanisms by which radiation-specific immune cells CD8+ T cells regulate gene expression in epithelial cells through the CCL5-SCD4 axis and B2M-TFRC to promote iron death in epithelial cells.

We propose that CD8+ T cells are central to intercellular communication in the dysregulated radiation immune microenvironment. To explore possible mechanisms by which radiation-specific CD8+ T cells may mediate the adverse effects induced by RT, we analyzed the abundance of radiation-specific immune cells in the three peripheral blood datasets described above and found that the abundance of radiation-specific immune cells was significantly increased after radiation compared to controls (**Figure 5A**). Similarly, we constructed intercellular communication networks in the validation set, and iTALK analysis identified significant differences in the abundance of ligand-receptor pairs in CD8+ T cells, endothelial cells, epithelial cells, and fibroblasts (**Figure 5B**). The enrichment of DEGs in each cell type in pathways involved in autophagy, apoptosis, ferroptosis and inflammatory bowel disease (**Figure 5C**), and in particular, radiation-specific CD8+ T cells activated the iron death pathway (**Figure 5D**), and these results drew our attention to our hypothesis that activation of radiation-specific CD8+ T cells by RT mediates iron death in intestinal epithelial cells.

In support of this preliminary analysis, the negative docking energies of CCL5 and SCD4 and B2M and TFRC were predicted by molecular docking (**Figure 5E**), suggesting that such ligand-receptor complexes can be formed *in vivo*. Based on these results, we hypothesized that radiation-specific immune cells CD8+ T cells regulate gene expression in epithelial cells through the CCL5-SCD4 axis and B2M-TFRC axis to promote ferroptosis in epithelial cells (**Figure 5F**).

## Discussion

While RT is known to affect the tumor microenvironment, including tumor blood vessels and immune system cells (16–19), how it triggers systemic bystander effects is unclear. Previous studies have implicated apoptosis and p53 signaling in such effects (20). Through preliminary analysis of single-cell data, we provide evidence that the immune microenvironment of tumors varies during irradiation, and that radiation-specific CD8+ T cells may be the primary risk factor for RT-associated adverse reactions.

Unlike targeted therapy, RT can promote phenotypic changes in normal interstitial cells and immune cells, leading to molecular and physiological changes in the microenvironment within the tumor (21–23). RT also spreads inflammatory signals, which reshape the immune environment within and around the tumor (24). These changes can affect the efficacy of anti-tumor immunotherapy (25), and they can lead to systemic bystander effects. Our results show that 1 day after radiation (T1), the abundance of both inflammatory and non-inflammatory immune cells next to the radiation focus is significantly reduced and the body’s immune system is suppressed, indicating that radiation therapy decreases the immune function of patients. At this time, radiation-specific CD8+ T cells were produced. Three days after radiation (T3), the abundance of CD4+ T cells, which suppress inflammation, increases significantly, and fibroblasts continue the high level of T1, suggesting that the body is in an immunosuppressive healing process at this time. At 7 days post-irradiation (T7), the abundance of inflammatory and non-inflammatory immune cells decreased again and was replaced by an active proliferation of epithelial cells, indicating that the organism was in a rapidly repairing hypoimmune state. Fourteen days after radiation (T14), the abundance of non-immune cells such as epithelial cells, endothelial cells, fibroblasts and stromal cells was almost unchanged compared to the healthy state (T0), while the significant change was in immune cells, suggesting that the effect of radiation therapy on the patient’s post-rehabilitation was concentrated on the immune cell ecosystem. Among them, the abundance of CD4+ T cells and monocytes significantly decreased, CD8+ T cells significantly increased, and B cells did not change significantly. Specifically, the only radiation-specific immune cells retained by the organism after patients recovered from radiation injury were radiation-specific CD8+ T cells, indicating that radiation-specific CD8+ T cells are a major risk factor for radiation-mediated systemic bystander effects. Unlike targeted therapies, radiation to tumor lesions can induce phenotypic changes in normal mesenchymal and cancer cells, leading to molecular and physiological alterations within the tumor microenvironment. These environmental modulations directly affect the degree of immunogenicity of the tumor microenvironment and may ultimately influence tumor responsiveness to tumor immunotherapy (25). And it was found that the propagation of inflammatory signals generated by RT reshapes the immune environment of the tumor microenvironment (24), thus suggesting that IR can reprogram the body’s immune microenvironment. In addition, due to the presence of RIBE, even unirradiated distant lesions showed the effect of RT response, suggesting that RT may also affect the tumor immune response, mainly by (1) inducing “immune cell death” and releasing tumor-associated antigens, (2) altering the immune phenotype of tumor cells, and (3) modulating the tumor microenvironment (21–23). Taken together, it can be shown that IR is able to mediate systemic bystander effects by reprogramming the body’s immune cells. Our results here suggest that RT promotes bystander effects by increasing the levels of radiation-specific CD8+ T cells, which activate inflammation-related pathways in endothelial cells, epithelial cells and fibroblasts through several genes, including CCL5, UBA52, CALM1, GNAI2, and GZMB.

Intercellular communication, mediated by ligand-receptor interactions, is the main way that immune cells kill their targets. Ligands can bind to specific cell surface receptors to initiate intracellular signal cascades, leading to various cellular responses, and the receptor-ligand axis can mediate the development of disease (26). For example, the phagocytic function of macrophages can be triggered by a variety of receptor-ligand interactions to eliminate pathogens and dead cells from the host (27). The recognition and killing of melanoma cells by natural killer (NK) cells is also controlled by a variety of activating receptor-ligand interactions (28). Based on the present study, we hypothesized that radiation-specific CD8+ T cells could establish communication with epithelial cells through the CCL5-SCD4 axis and the B2M-TFRC axis. Subsequently, ferroptosis of epithelial cells is promoted. Ferroptosis is an iron-dependent, new type of programmed cell death that is different from apoptosis, cell necrosis, and autophagy (29, 30). Unlike apoptosis, which is immunologically silent, ferroptosis is immunogenic: the affected cells release damage-associated molecular patterns and alarm elements, amplifying cell death and promoting a series of inflammation-related reactions (31–33). The CCL5-mediated receptor-ligand axis has been shown to be a potential therapeutic target for radiation pulmonary toxicity (34) and is associated with iron death (35). The current study extends this literature by elucidating other ligand-receptor axes involved in post-RT radiation-specific CD8+ T cells.

In summary, our research suggests that RT generates radiation-specific immune cell subsets, which reprogram the immune ecosystem and mediate systemic bystander effects. These radiation-specific immune cells, particularly CD8+ T cells, participate in a wide range of cell-to-cell communication events. These cells persist *in vivo* after RT and may mediate the development of adverse reactions. In conclusion, our study provides key information on the heterogeneous response of cancer patients to RT. However, there are limitations to our study and our bioinformatic analysis should be verified and extended in biological experiments, which will bring us closer to making RT safer and more effective.

## Method

### Data processing

10×Genomics single-cell RNA sequencing data GSE165318 derived from Mus musculus, containing results from irradiated intestinal tissues, was obtained from the GPL 21493 platform of the Gene Expression Omnibus (GEO) database (36). Data corresponded to day 1 (T1 state, including four samples), day 3 (T3 state, including three samples), day 7 (T7 state, including three samples), and day 14 (T14 state, including three samples) after irradiation. Data also corresponded to unexposed healthy intestinal tissue (T0 state, including four samples). Additionally, gene expression profile of cells from human irradiated peripheral blood were also taken from the GSE21240 database (48 samples) on the GPL6480 platform, GSE55953 database (28 samples) on the GPL14550 platform, and GSE65292 database (35 samples) on the GPL13497 platform. These data were used to further verify the expression of the markers in human species.

### Quality control of single-cell RNA sequencing and cell cluster analysis

The Seurat package (37) was used for quality control (QC) of single-cell RNA sequencing data and cell cluster analysis. The original expression profile of GSE165318 contains a total of 19,588 genes and 22,680 cells. The default QC standard is to screen out genes detected in <3 cells. In addition, cells with more than 6,000 or less than 200 genes expressed in the cells and more than 10% of low-quality cells extracted from the mitochondrial genome will also be discarded. After QC, a total of 18.668 genes and 22,585 cells were obtained for subsequent analysis.

Moreover, the “FindAllMarkers” function of Seurat package was used to determine the marker of the cluster, and cell type was determined according to the marker. The t-distributed stochastic neighbor embedding (t-SNE) method (38) was used to visualize the results of cell cluster analysis.

### Pseudo-time analysis

Pseudo-time analysis can infer the differentiation trajectory of immune cells in irradiated intestinal tissues of mice or the evolution of cell subtypes during development. Monocle 3 in R (39) was used to organize cells into potentially discontinuous trajectories and then perform statistical tests to identify genes differentially expressed on the trajectories find different genes expressed on these trajectories. This pseudo-time analysis was visualized in three dimensions using the Monocle 3 workflow. The Uniform Manifold Approximation and Projection (UMAP) method (40) was used to visualize the results.

### Intercellular communication analysis

Ligand-receptor binding is one of the main forms of signal transduction between neighboring and distant cells. In this study, for immune cells dysregulated in irradiated intestinal tissues of mice, we used the freely available R package iTALK (41) for cell interaction analyses. Take the cell gene expression matrix in scRNA-seq as input, generate a sorted gene list through iTalk to identify highly expressed ligand-receptor pairs, and then use the iTalk database to search and pair ligands and receptors, and visualize the results as circos plot. The ligand-receptor relationships are based on data from a built-in database for iTALK.

### Gene regulatory networks analysis

Single-cell regulatory network inference and clustering (SCENIC) is a tool to infer gene regulatory network and related cell status based on single-cell RNA-seq data (42). In this study, we comprehensively reconstructed the transcription factor-centered gene regulatory network to further explore the regulatory mechanisms of dysregulated immune cells in irradiated intestinal tissues of mice. We used PySCENIC (43) (a lightning-fast python implementation of the SCENIC pipeline) to analyze networks of gene expression based on regulation by transcription factors. There are three steps in total. First, the per-target regression method (GRNBoost 2) is used to infer the co-expression module. Next, use cis-regulatory motif discovery (CisTarget) to prun off indirect targets from these modules. Finally, quantify the activity of these regulators by scoring the enrichment of the regulator target gene (AUcell). After that, save the results obtained in pySCENIC into.loom format, import them into the R environment, and visualize the results.

### Molecular docking

Molecular docking was performed using Hex software 8.0.0 to predict ligand-receptor binding relationships in different cells (44). Complexes with docking energy < 0 were considered as possible binding interactions. In addition, 3D models of docking domains were visualized by Pymol software (45).

### Recipient operating characteristic curves and functional richness

We evaluated the ability of cellular marker genes to diagnose radiation-induced adverse reactions by using the pROC software package (46) to generate ROC curves. The clusterProfiler package was used to examine the functional enrichment of genes differentially expressed in response to radiation (47). p < 0.05 was considered as a significant enrichment.

### Data analysis and statistics

All bioinformatic analyses in this study were performed based on the Bioinforcloud platform (http://www.bioinforcloud.org.cn).

## Data availability statement

The datasets presented in this study were found in online repositories. The names of the repositories and accession numbers can be found in the article/supplementary material. Further enquiries should be directed to the corresponding author.

## Author contributions

SM and YL designed the study. XH and QS performed the analysis and wrote the manuscript. TL, YC, NZ, CH, GH, and MW participated in the analysis and reviewed the manuscript draft. GH was mainly responsible for the guidance of radiology. All authors contributed to the article and approved the submitted version.

## Acknowledgments

We would like to thank the patients and investigators who participated in the TCGA and the GEO for providing data.

## Conflict of interest

The authors declare that the research was conducted in the absence of any commercial or financial relationships that could be construed as a potential conflict of interest.

## Publisher’s note

All claims expressed in this article are solely those of the authors and do not necessarily represent those of their affiliated organizations, or those of the publisher, the editors and the reviewers. Any product that may be evaluated in this article, or claim that may be made by its manufacturer, is not guaranteed or endorsed by the publisher.
